# On Calisaya Extract

**Published:** 1848-02

**Authors:** Charles Ellis


*On Calisaya Extract.* By CHARLES ELLIS.—The object of this notice is to induce an examination and trial of a preparation from the Calisaya Bark, which may perhaps be found to be a valuable addition to the list of medicinal agents.

The residuary product left after the crystallization of sulphate of quinia, known by the name of impure sulphate of quinia, extract of quinia, and precipitated extract of bark, has long been employed in this country as a remedy in intermittent fevers. It is analogous to chinoidine and contains amorphous quinine, an interesting article upon which, from the pen of Baron Liebig, was published in Vol. 18, No. 3, of the

American Journal of Pharmacy. According to this writer, the chief constituent of chinoidine bears the same relation to quinia that uncrystalline sugar does to crystalline, in fact that it is amorphous quinine. These facts are sufficient to show that the calisaya bark (Cinchona flava) has other constituents, besides its crystallizable alkaloids, of too much importance to be overlooked.

The article under notice, containing as it does, all the quinia and cinchonia in the bark, must necessarily be a more valuable preparation, than one from which those important vegetable alkalies have been separated by crystallization, so far as is usually the case in the manufacture of sulphate of quinia.

The frequent demand for an extract of calisaya bark, upon which reliance may be placed when administered in small doses, and the preference often given to it by physicians, over the sulphate of quinia, are sufficient reasons, without any claim to novelty, for introducing such a preparation through the pages of our Journal. In order to distinguish it from the officinal *Extract. Cinchonæ*, as well as from the residuary product alluded to above, the name of Calisaya Extract (Extractum Calisayacum) is proposed.

The following is the mode of preparing it:

The Calisaya Bark (Cinchona flava) finely bruised two pounds, boil in a gallon of water acidulated with f.℥ss. Acid Hydrochloric; strain and boil the residue in two successive portions of water, of one gallon each, with a similar quantity of acid. To these decoctions mixed together, add about ℥ij. or a sufficient quantity of lime, previously reduced to powder by the addition of water. Stir the mixture well, and set it aside until precipitation ceases to take place.

This precipitate being strained off, is to be well washed with water, dried, and digested in hot alcohol, until all the taste has been exhausted; then distil off the alcohol, and carefully evaporate the product over a water bath to a pillular consistence.

This extract will contain the quinia, the cinchona, and all that can possess any medical value in the bark. It will be found to be intensely bitter, and if a selection of the true calisaya bark has been made, it is believed that the product thus obtained, will prove a very efficient remedy, in doses but little larger than those in which the sulphate of quinia is usually administered.

The proximate principles of the bark will remain in this preparation, in the state of alkaloids, uncombined with acid which is thought preferable for the more common mode of administration in pills. If a solution is wanted, the addition of a few drops of sulphuric acid will assist in rendering it entirely soluble in water, and will convert the vegetable alkalies into sulphates.

It may be given in doses of from one to four grains; medium dose two grains, and is applicable to the same character and form of disease in which bark in substance, and the sulphate of quinia, are usually administered.—*American Journal of Pharmacy.*

*On preparing pure Cubebin.* By EMIL NOLLENBERGER. Apothecary, U. S. Naval Hospital, New York.—Wishing to prepare pure Cubebin in large quantities, I tried many experiments to find out the cheapest and most practical mode. The following I found to answer best.

The cubebs are first coarsely powdered and macerated with a sufficient quantity of water in a copper still for twenty-four hours, then distilled as long as volatile oil passes over. After drying the residue, it is exhausted with alcohol, spec. gr. 0.85 ; the tincture is then put in a still and the alcohol driven over. The resinous matter which remains in the still is put aside for several days, till it forms a mass of crystals, which are placed on a strainer where the soft resin is almost entirely separated. The remainder on the strainer is dissolved in three or four times its weight of boiling alcohol, spec. gr. 0.90, and the supernatant portion decanted while warm, from the precipitated resin, when upon cooling the cubebin crystallizes. By dissolving it again in hot strong alcohol and filtering it through animal charcoal, it is obtained in beautiful white, needle-shaped crystals.—*Ibid.*

